# Supplementary material for: Massively parallel profiling of RNA-targeting CRISPR-Cas13d
Source: Nat Commun. 2024 Jan 12;15:498. doi: 10.1038/s41467-024-44738-w (PMC10786891; doi:10.1038/s41467-024-44738-w)
Supplement: Supplementary file 1 — Supplementary Information [file 41467_2024_44738_MOESM1_ESM.pdf]

SUPPLEMENTARY FIGURES

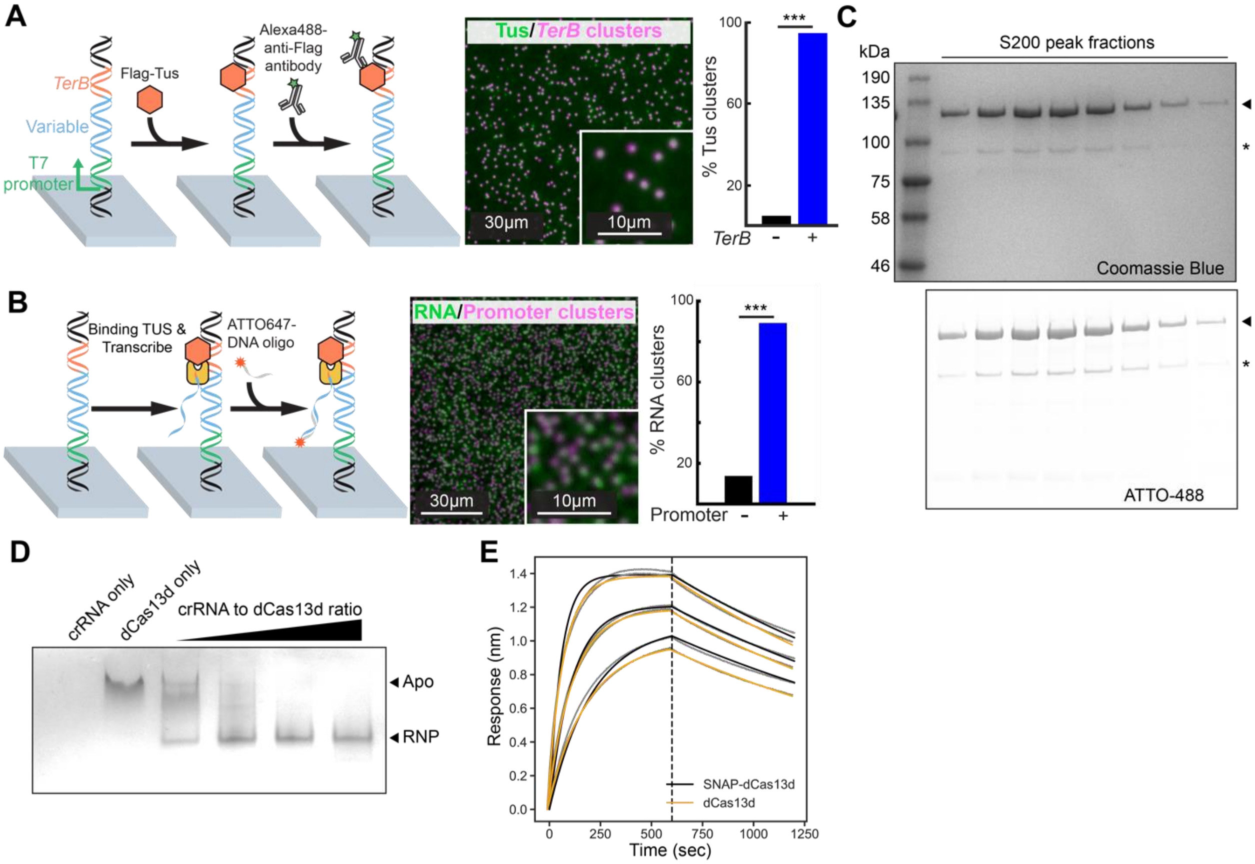

Figure S1. RNA-CHAMP: A platform for massively parallel measurement of Cas13d binding. (A) Left: schematic of the experiment. Middle: microscope field-of-view. Right: quantification of fluorescent Tus (green) binding to *TerB*-containing DNA clusters (magenta) in a MiSeq chip. The bar graph shows the percentage of clusters with or without *TerB*-encoding sequences that also have a fluorescent Tus signal. Unpaired Student's t-test, $^{***}p < 0.001$. **(B)** Left: schematic of the experiment, Middle: microscope field-of-view. Right: quantification of RNA transcription in a MiSeq chip. The bar graph shows the percentage of clusters that hybridized with an ATTO-647N labeled complementary oligo (green) to promoter containing clusters (magenta) and clusters without a T7 promoter. Unpaired Student's t-test, $^{***}p < 0.001$. **(C)** Purification of ATTO-488 labeled SNAP-dCas13d. Fractions were collected from an S200 gel filtration column and resolved on an SDS-PAGE gel. Top: gel visualized by Coomassie blue staining. Bottom: fluorescent image before Coomassie blue staining. Triangle: ATTO-488 labeled SNAP-dCas13d; *: minor truncation product. **(D)** Purification of ATTO-488 labeled dCas13d conjugated with a CRISPR RNA (crRNA). Purified dCas13d was incubated with various amounts of crRNA. The crRNA:dCas13d binary complex was separated from apoCas13d via size exclusion chromatography and analyzed in a native tris-glycine gel followed by Coomassie blue staining. **(E)** BLI results of SNAP-dCas13d and non-tagged dCas13d. The SNAP-tag does not affect Cas13d's binding affinity to RNA.

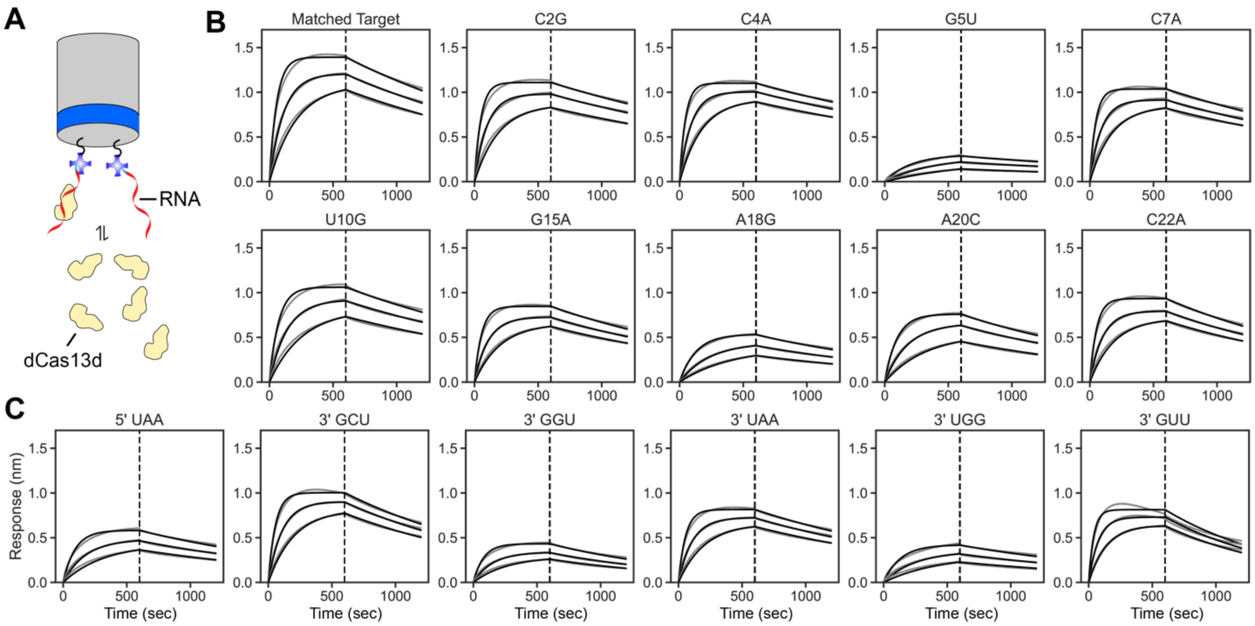

Figure S2. Biolayer interferometry (BLI) of a subset of the target RNA library.
(A) Schematic of the BLI assay. Biotinylated target RNA is immobilized on a streptavidin-functionalized BLI tip. Separate tips are used for each target RNA. Tips are dipped into a solution containing dCas13d RNPs to monitor the association rate, k_a. Dissociation, k_d, is monitored by transferring the tip to a buffer solution without any free RNP. For each target sequence, all measurements were repeated with 100 nM, 50 nM, and 25 nM dCas13d. BLI results for **(B)** partially matched and **(C)** PFS sequences. RNA-loaded biosensors were immersed in dCas13d solutions for 600 sec (dashed vertical line). Biosensors were then immersed in the binding buffer for k_d measurements. Black lines are a global simultaneous fit to three concentrations. Gray lines are raw BLI curves. Fit results are summarized in **Table S1**.

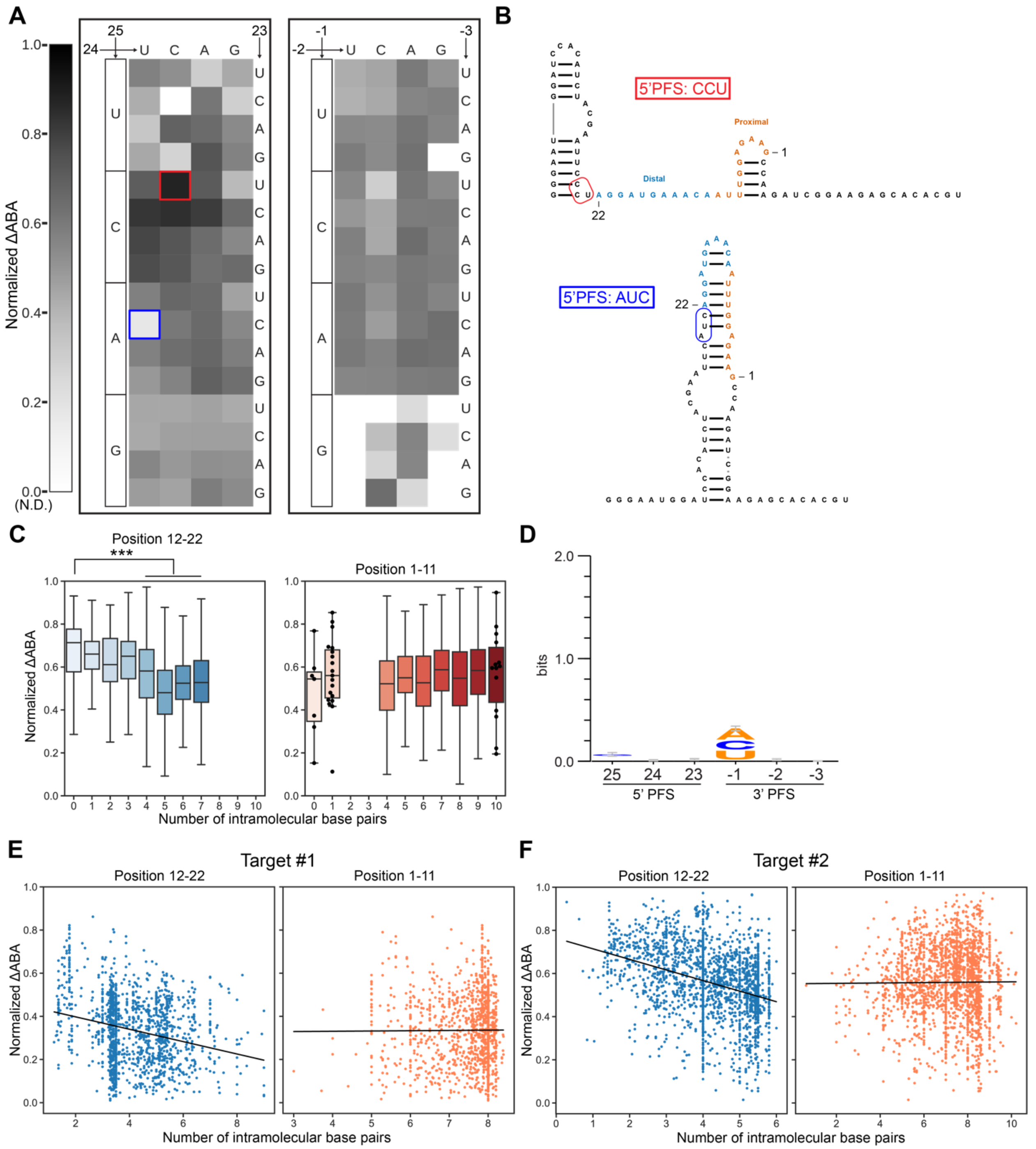

Figure S3. Cas13d PFS binding analysis on a second target RNA library.

(A) Normalized ΔABAs of Cas13d binding to a PFS library with a second target RNA (left: 5' PFS; right: 3' PFS). Blue and red boxes indicate two targets that are highlighted in the next panel. These sequences have a matched target RNA but significant changes in binding affinities. **(B)** The ViennaRNA-predicted secondary structures of 5'PFS-CCU (red box) and 5'PFS-AUC (blue box). Light blue indicates the target RNA sequence. **(C)** Normalized ΔABA of PFS

sequences grouped by their intramolecular base pairing counts in the target region. Left: intramolecular base pairing counts in positions 12-22; right: positions 1-11 of the target RNA. Error bars are the standard deviation of normalized ΔABA. Statistical analysis was performed using unpaired Student's t-test, $***p < 0.001$. **(D)** Sequence logo of the 25% highest affinity PFS sequences across two target RNA libraries from **Figures 2A** & **S3A**. **(E)** Scatter plot of the average number of intramolecular base pairs and normalized ΔABA for target #1. Left: distal positions 12-22 (Pearson's $r = -0.25$, p-value < 0.0001). Right: proximal positions 1-11 (Pearson's $r = 0.0067$, p-value = 0.80). **(F)** Scatter plot of the average number of intramolecular base pairing and normalized ΔABA for target #2. Left: distal positions 12-22 (Pearson's $r = -0.35$, p-value < 0.0001). Right: proximal positions 1-11 (Pearson's $r = 0.0080$, p-value = 0.72)

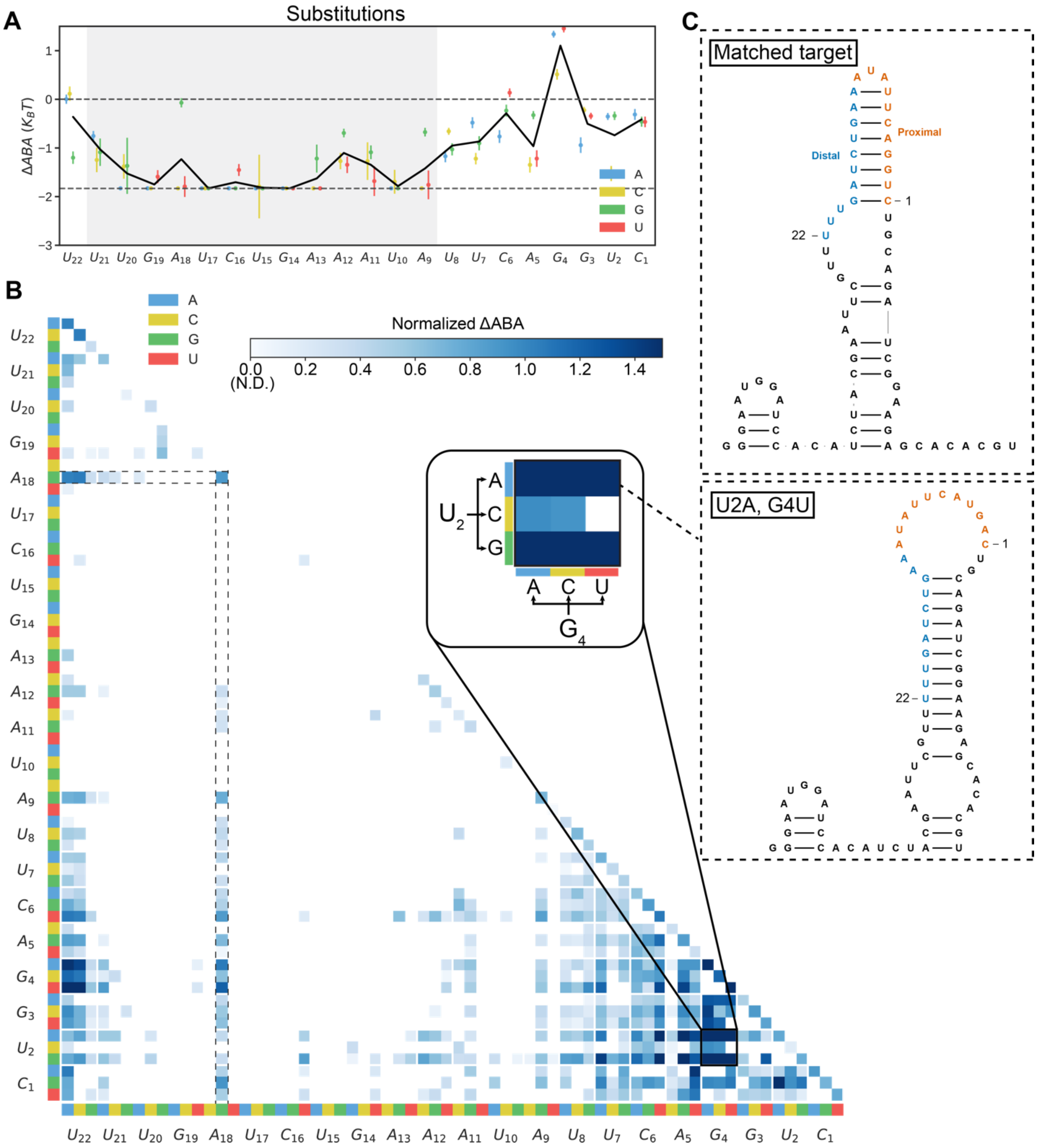

Figure S4. Mismatch analysis of a second target RNA library.
(A) Summary of single mismatch-dependent changes in the ΔABA for the second target RNA. The solid black line is the average of all possible substitutions at each position. The upper dashed line is the matched target ΔABA and the lower dashed line is the RNA-CHAMP detection limit. **(B)** The normalized ΔABA for all double substitutions (normalized to the matched target). Inset: blowup of all mismatches at target positions U_2 & G_4. N.D. (not determined) refers to sequences

that bound Cas13d with a lower affinity than our detection limit. **(C)** Substitutions that increase intramolecular base pairing drastically increased the ΔABA. Top: predicted matched target RNA structure. Bottom: predicted U2A, G4U structure. Despite two mismatches, this partially matched target binds Cas13d better than the matched target due to relaxed intramolecular base pairing.

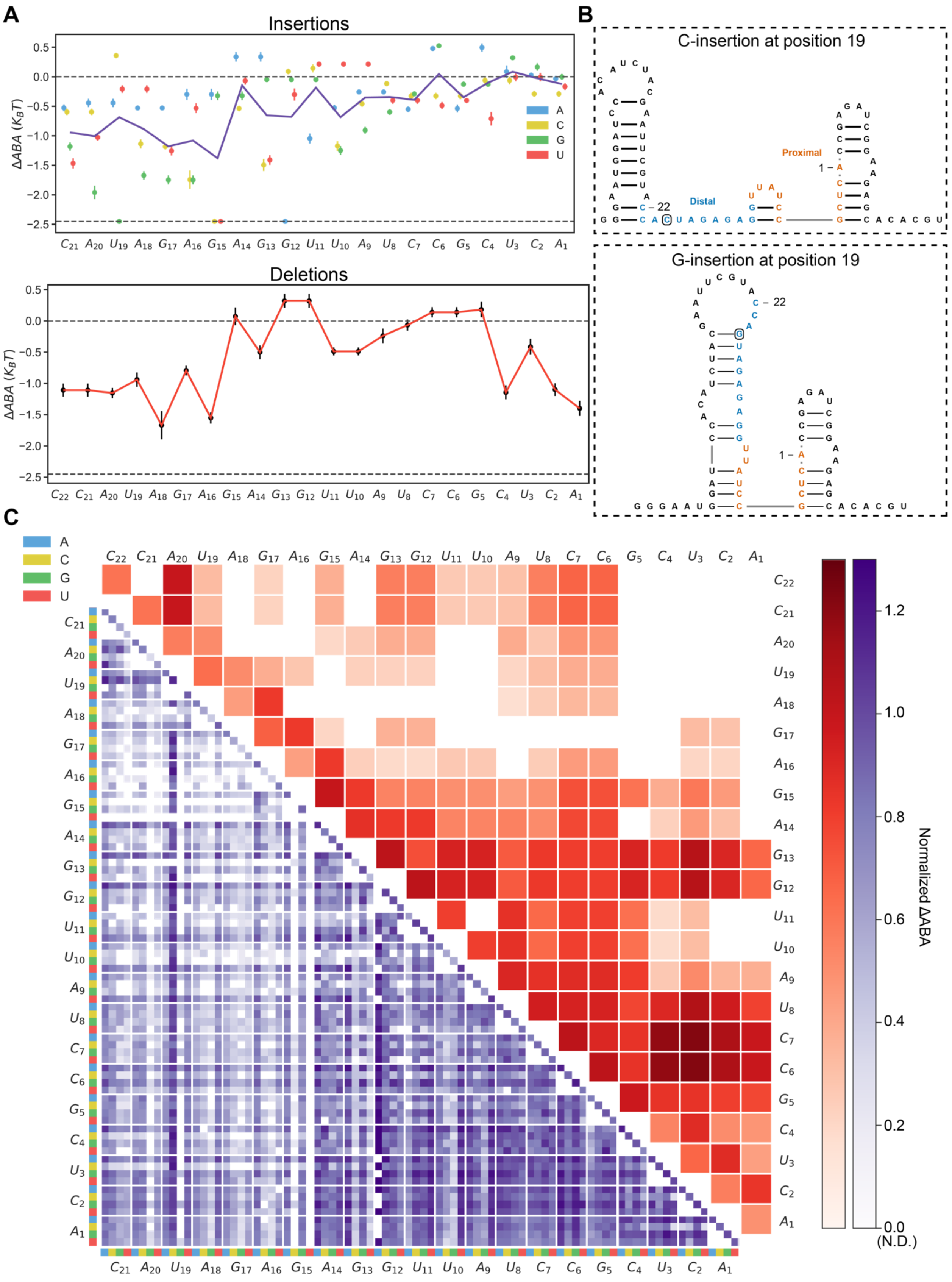
A
Insertions
ΔABA (K_BT)
A
C
G
U
Deletions
ΔABA (K_BT)
B
C-insertion at position 19
Distal
Proximal
G-insertion at position 19
C
Normalized ΔABA
(N.D.)

Figure S5. Insertion and deletion analysis for the first target.
(A) Changes in the ΔABA for all possible insertion (top) and deletion (bottom). For insertions, the line is an average of the four possible insertions at each position. The upper dashed line is the matched target ΔABA and the lower dashed line is the RNA-CHAMP detection limit. **(B)** Predicted structures of two insertions at position 19 that relax (19C, top) or further basepair (19G, bottom) with the target RNA. These changes have a drastic impact on the ΔABA. **(C)** Top triangle plot: double deletion analysis. Bottom triangle plot: double insertion analysis.

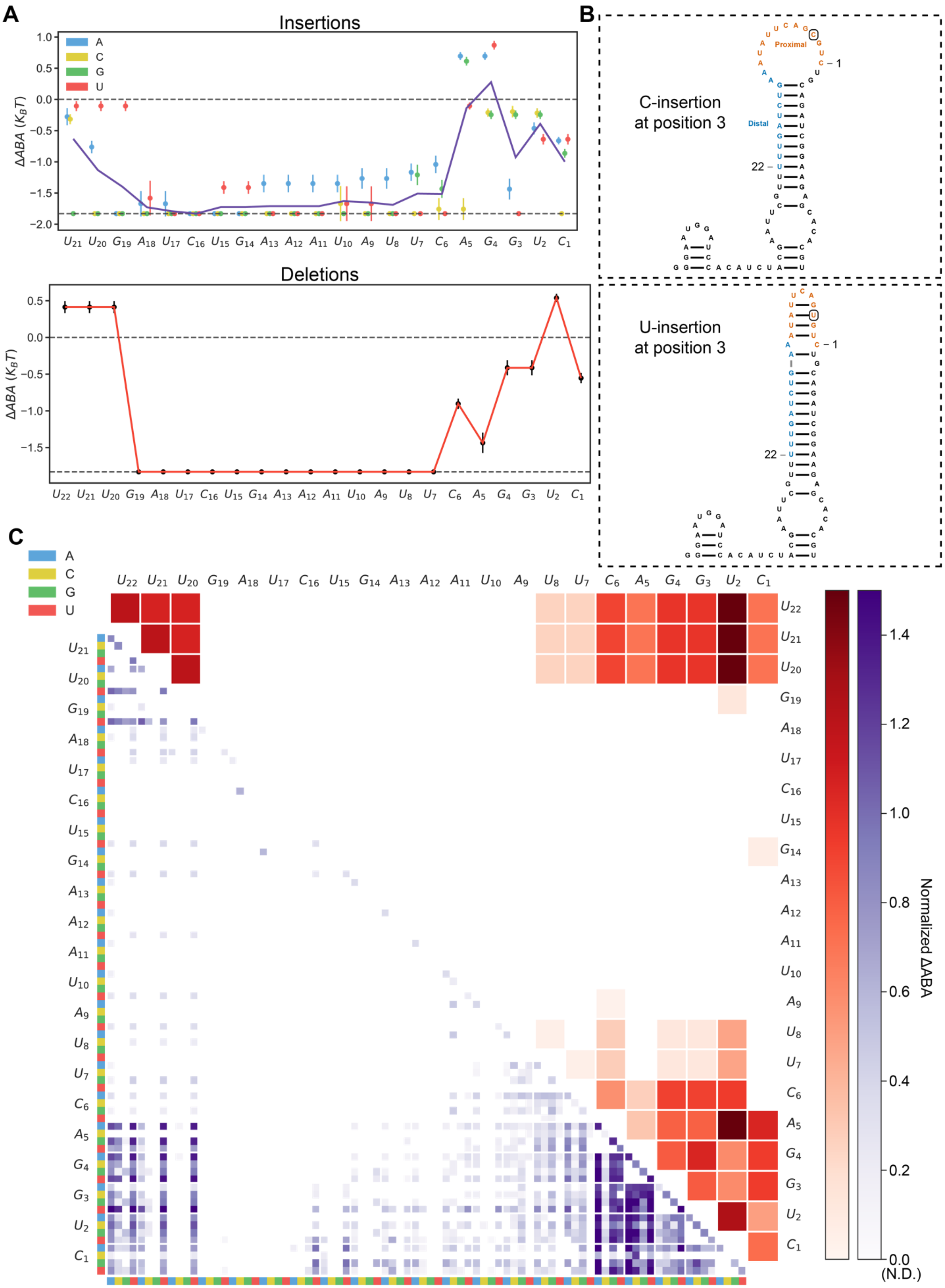
A
Insertions
ΔABA (K_BT)
A
C
G
U
Deletions
B
C-insertion at position 3
Proximal
Distal
U-insertion at position 3
C
Normalized ΔABA
0.0 (N.D.)

Figure S6. Insertion and deletion analysis for a second target.
(A) Changes in the ΔABA for all possible insertion (top) and deletion (bottom). For insertions, the line is an average of the four possible insertions at each position. The upper dashed line is the matched target ΔABA and the lower dashed line is the RNA-CHAMP detection limit. **(B)** Predicted structure of two insertions at position 3 that relax (3C, top) or further basepair (3U, bottom) with the target RNA. **(C)** Top triangle plot: double deletion analysis. Bottom triangle plot: double insertion analysis.

A

Matched target: CCATAGAGAGGTTATCCGCTCA
Encoding sequence: CUATAGAGAGGTTATCCGGTCA

Position 4 substitution-G, Position 21 sustitution-U

Position	Substitution A	Substitution U	Substitution G	Substitution C	Insertion A	Insertion U	Insertion G	Insertion C	Deletion
1	0	0	0	0	0	0	0	0	0
2	0	0	0	0	0	0	0	0	0
3	0	0	0	0	0	0	0	0	0
4	0	0	1	0	0	0	0	0	0
⋮									
21	0	1	0	0	0	0	0	0	0
22	0	0	0	0	0	0	0	0	0

B

Encoding sequence: CUGTAGAGAGGTTATCCGCTCA

C21U, A20G

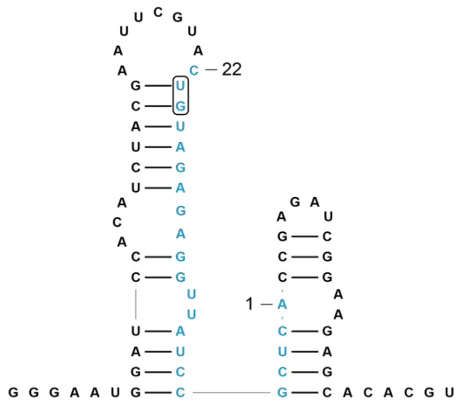

Position	Base pairing
1	0
2	1
3	1
4	1
5	1
6	1
7	1
8	1
9	1
10	0
11	0
12	1
13	1
14	0
15	0
16	1
17	1
18	1
19	1
20	1
21	1
22	0

C

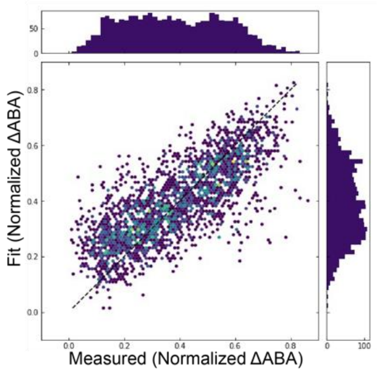

Figure S7. Cas13d modeling and machine learning model.
(A) Each position along the target has eight possible alterations including substitutions, insertions, and deletions. For example, position four, which is a cytosine in the matched target, can be substituted for A, U, or G (C4A, C4U, or C4G), can have one of four insertions (A, U, G, C), or can be deleted. **(B)** Target RNA accessibility is encoded as the number of intramolecularly base paired nucleotides, as illustrated. RNA structure is predicted by the ViennaRNA package.

(C) We trained half of our dataset on a convolutional neural network (CNN) and tested the rest of the dataset with a training alteration of 1,000 (epoch). In this machine learning model, there are 37,089 total trained parameters. Pearson's $r = 0.77$.

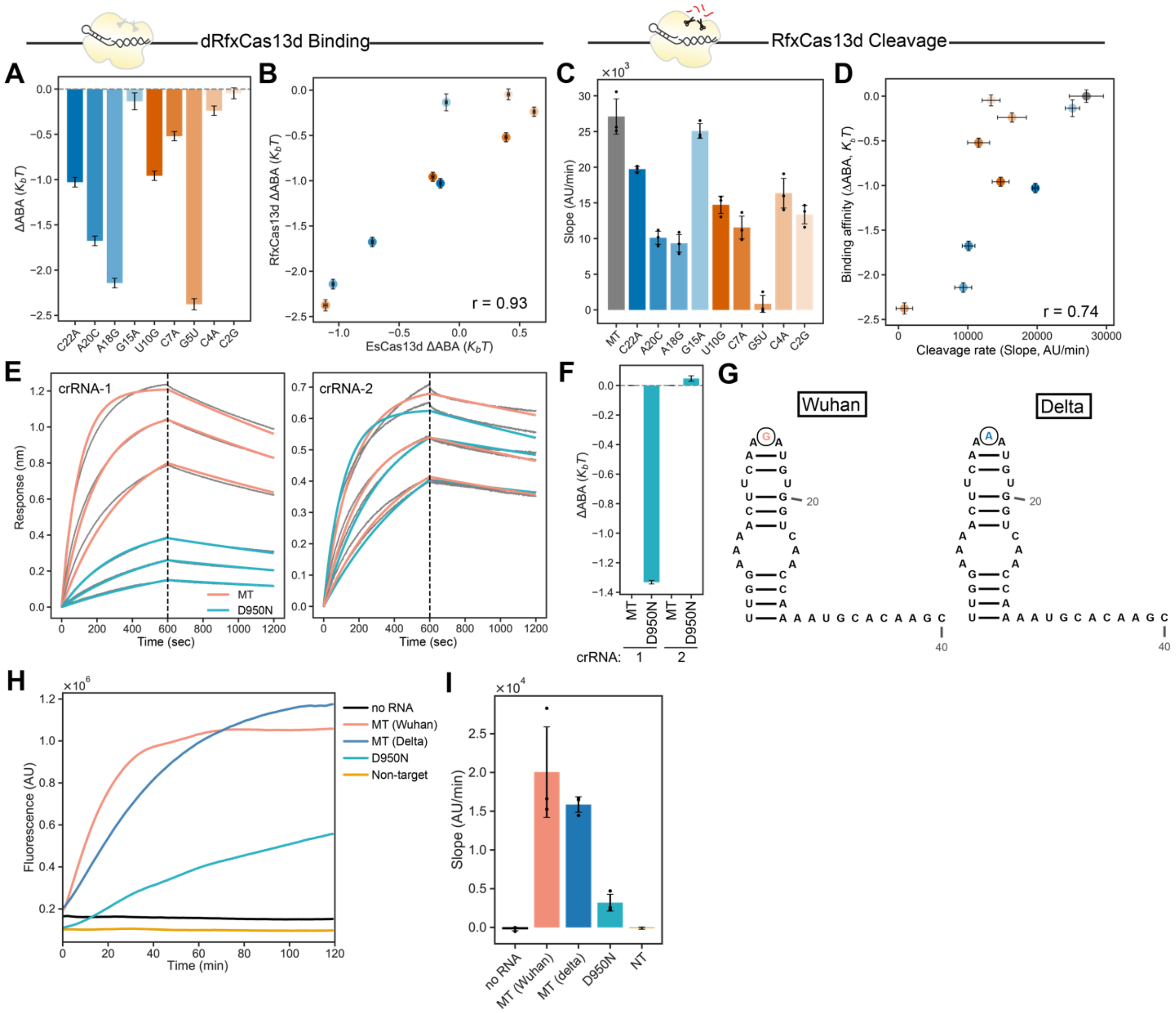

Figure S8. Binding and cleavage sensitivity of Cas13d for CRISPR diagnostics. (A) Relative DNA binding affinities for nuclease-dead RfxCas13d, as measured by biolayer interferometry (BLI). Target RNA sequences harbor the indicated single mismatches relative to the matched target RNA. Binding affinities are normalized to the matched target. Error bars: S.D. of three replicates. Blue: proximal mismatches; red: distal mismatches. **(B)** Correlation of ΔABAs of dEsCas13d and dRfxCas13d. Pearson r = 0.93, p-value < 0.001. **(C)** Cleavage rates for the indicated mismatched targets, defined as the slope of the first 20 minutes of the fluorescent reporter assay (see Methods). **(D)** Correlation of binding affinity and cleavage rates for RfxCas13. Pearson r = 0.74, p-value < 0.05. When the three proximal mismatches — C2G, C4A, and C7A — are excluded, Pearson r = 0.95 (p-value < 0.05). **(E)** BLI curves of crRNA-1 and crRNA-2 with MT and D950N target RNAs. Colored lines are a global fit to three concentrations. Gray lines: measured BLI binding curves. **(F)** The apparent binding affinity is computed from a global fit to the data. **(G)** A single C→A substitution differentiates the Wuhan and Delta strains (circled). Notably, this substitution is not predicted to change the target RNA structure. **(H)** Time traces of the fluorescent signal for each of the indicated target RNA

sequences. **(I)** Initial slope of the fluorescent signals in (H), computed up to the 20-min time point.

Supplemental Tables

Table S1. Biolayer interferometry and RNA cleavage rates for select RNAs

Sequences	K_d (M)	K_d Error	k_a ($M^{-1}S^{-1}$)	k_a Error	k_d (S^{-1})	k_d Error	Numbers of sequences for RNA-CHAMP in Fig 1G
MT	3.1E-09	1.8E-11	1.7E+05	6.4E+02	5.2E-04	2.5E-06	26270
C2G	2.0E-09	1.6E-11	2.0E+05	8.1E+02	4.0E-04	2.6E-06	58
C4A	1.6E-09	1.0E-11	2.2E+05	6.6E+02	3.5E-04	1.9E-06	77
G5U	9.3E-09	1.2E-10	4.5E+04	3.5E+02	4.2E-04	4.1E-06	73
C7A	2.1E-09	1.3E-11	2.2E+05	7.9E+02	4.5E-04	2.3E-06	72
U10G	3.8E-09	2.9E-11	1.3E+05	6.3E+02	5.1E-04	3.1E-06	85
G15A	3.4E-09	1.9E-11	1.8E+05	6.4E+02	6.0E-04	2.4E-06	93
A18G	8.7E-09	4.8E-11	7.3E+04	2.8E+02	6.3E-04	2.5E-06	89
A20C	6.3E-09	3.4E-11	10.0E+04	3.7E+02	6.3E-04	2.5E-06	85
C22A	3.6E-09	2.0E-11	1.9E+05	7.2E+02	6.6E-04	2.6E-06	77
5’-UAA	6.0E-09	5.4E-11	1.0E+05	6.2E+02	6.1E-04	4.0E-06	33
3’-GCU	4.2E-09	2.0E-11	1.7E+05	5.9E+02	7.2E-04	2.3E-06	46
3’-GGU	7.1E-09	5.8E-11	1.2E+05	7.3E+02	8.4E-04	4.3E-06	98
3’-UAA	6.0E-09	5.4E-11	1.0E+05	6.2E+02	6.1E-04	4.0E-06	22
3’-UGG	8.6E-09	1.1E-10	7.0E+04	6.0E+02	6.0E-04	5.4E-06	92
3’-GUU	4.4E-09	3.5E-11	2.5E+05	1.6E+03	1.1E-03	4.7E-06	99
crRNA-1 (MT)	4.0E-09	3.8E-11	9.6E+04	4.7E+02	3.8E-04	3.1E-06	
crRNA-1 (D950N)	1.5E-08	1.1E-10	2.7E+04	1.5E+02	4.1E-04	2.2E-06	
crRNA-2 (MT)	3.0E-09	3.8E-11	5.9E+04	2.3E+02	1.8E-04	2.2E-06	
crRNA-2 (D950N)	2.9E-09	3.8E-11	8.7E+04	4.3E+02	2.5E-04	3.0E-06	

Table S2. Slope of fluorescent cleavage assay for selected RNAs

Sequences	Normalized slope	S.D.
MT	1	N/A
C22A	0.57	0.048
A20C	0.69	0.082
A18G	0.54	0.078
G15A	0.71	0.030
U11A	0.71	0.017
U10A	0.17	0.028
U10G	0.37	0.0059
A9U	0.48	0.028
U8A	0.19	0.020
C7A	0.15	0.021
C7G	0.38	0.0087
C6G	0.077	0.013
G5U	0.040	0.0080
G5C	-0.055	0.085
C4A	0.027	0.00095
C4G	0.12	0.030
U3A	-0.013	0.0050
C2G	0.026	0.0053
A1U	-0.020	0.046
**Sequences**	**Slope (AU/min)**	**S.D.**
crRNA-1 (MT)	20000	5800
crRNA-1 (D950N)	3200	1100
crRNA-2 (MT)	23000	820
crRNA-2 (D950N)	4700	550

Table S3. DNA oligonucleotides used in this study

Name	Type	Description	Sequence (5'-3')
Library extension primer- Forward (JK044)	Oligo	Extend library oligo with Illumina adapters	AATGATACGGCGACCACCGAGATCTACACTCTTTCCCTACA CGACGCTCTTCCGATCT
Library extension primer- Reverse (JK045)	Oligo	Extend library oligo with Illumina adapters	CAAGCAGAAGACGGCATACGAGATGAACAACATGACGTG ACTTTAGTTACAACATACTAATTGTGACTGGAGTTCAGACG TGTGCTCTTCCGATCT
Target 1	Oligo pool	6N PFS oligo library	CCTACACGACGCTCTTCCGATCTTAATACGACTCACTATAG GGAATGGATCCACATCTACGAATTCNNNCCATAGAGAGGT TATCCGCTCANNNAGATCGGAAGAGCACACGTCTGAAC
Target 2	Oligo pool	6N PFS oligo library	CCTACACGACGCTCTTCCGATCTTAATACGACTCACTATAG GGAATGGATCCACATCTACGAATTCNNNGTTGTTCTCCGTC TATAAATACNNNAGATCGGAAGAGCACACGTCTGAAC
t7_promoter_scra mble	Oligo	T7 promoter scramble oligo	CCTACACGACGCTCTTCCGATCTACGGTAGATCTAAAGTCA CTAATGGATCCACATCTACGAATTCNNNTTTGATCTGAAAT ATTCAGGTCNNNAGATCGGAAGAGCACACGTCTGAAC
Non-target negative control	Oligo	Non-target oligo as a negative control	CCTACACGACGCTCTTCCGATCTTAATACGACTCACTATAG GGAATGGATCCACATCTACGAATTCGTTAGCTAGAAGGGG AAGTTGGTTATGGAGATCGGAAGAGCACACGTCTGAAC
P7 regeneration primer (IF363)	Oligo	Regenerate all clusters into dsDNA on the chip	CAAGCAGAAGACGGCATACGAGAT
PhiX labeling primer (IF443)	5'-Digoxigenin labeled Oligo	Regenerate PhiX clusters on the chip	/5DigN/CGGTCTCGGCATTCCTGCTGAACCGCTCTTCCGATC
T7 promoter_Forward	Oligo	Universal forward oligo for IVT template	TAATACGACTCACTATAGGG
A1U_Reverse	Oligo	Reverse oligo for IVT template	CGATCTCGGAGAGCGGATAACCTCTCTATGGTACGAACCC TATAGTGAGTCGTATTA
U3A_Reverse	Oligo	Reverse oligo for IVT template	CGATCTCGGTGTGCGGATAACCTCTCTATGGTACGAACCCT ATAGTGAGTCGTATTA
C4G_Reverse	Oligo	Reverse oligo for IVT template	CGATCTCGGTGACCGGATAACCTCTCTATGGTACGAACCCT ATAGTGAGTCGTATTA
G5C_Reverse	Oligo	Reverse oligo for IVT template	CGATCTCGGTGAGGGGATAACCTCTCTATGGTACGAACCCT ATAGTGAGTCGTATTA
C6G_Reverse	Oligo	Reverse oligo for IVT template	CGATCTCGGTGAGCCGATAACCTCTCTATGGTACGAACCCT ATAGTGAGTCGTATTA
C7G_Reverse	Oligo	Reverse oligo for IVT template	CGATCTCGGTGAGCGCATAACCTCTCTATGGTACGAACCCT ATAGTGAGTCGTATTA
U8A_Reverse	Oligo	Reverse oligo for IVT template	CGATCTCGGTGAGCGGTTAACCTCTCTATGGTACGAACCCT ATAGTGAGTCGTATTA
A9U_Reverse	Oligo	Reverse oligo for IVT template	CGATCTCGGTGAGCGGAAAACCTCTCTATGGTACGAACCC TATAGTGAGTCGTATTA
U10A_Reverse	Oligo	Reverse oligo for IVT template	CGATCTCGGTGAGCGGATTACCTCTCTATGGTACGAACCCT ATAGTGAGTCGTATTA

U11A_Reverse	Oligo	Reverse oligo for IVT template	CGATCTCGGTGAGCGGATATCCTCTCTATGGTACGAACCCTATAGTGAGTCGTATTA

Table S4. RNA oligonucleotides used in this study

Name	Type	Description	Sequence (5'-3')
crRNA_ Target 1	crRNA	PFS analysis and mismatch analysis	CACCCGUGCAAAAAUGCAGGGGUCUAAAACUGAGCGGAUA ACCUCUCUAUGG
crRNA_ Target 2	crRNA	PFS analysis	CACCCGUGCAAAAUUGCAGGGGUCUAAAACCUUCUCCAAA UUGUUUCAUCCU
crRNA_ Target 3	crRNA	Mismatch analysis	CACCCGUGCAAAAUUGCAGGGGUCUAAAACGACCUGAAUA UUUCAGAUCAAA
crRNA_Target 1 (RfxCas13d)	crRNA	BLI and fluorescent cleavage assay	AACCCCUACCAACUGGUCGGGGUUUGAAACUGAGCGGAUA ACCUCUCUAUGG
crRNA-1	crRNA	SNP detection (Fig 5)	CACCCGUGCAAAAUUGCAGGGGUCUAAAACUUUUGGUUGA CCACAUCUUGAA
crRNA-2	crRNA	SNP detection (Fig 5)	CACCCGUGCAAAAUUGCAGGGGUCUAAAACCUUGAAGUUU UCCAAGUGCACU
crRNA-1 (Delta)	crRNA	SNP detection (Fig. S8H)	CACCCGUGCAAAAUUGCAGGGGUCUAAAACUUUUGGUUGA CCACAUUUUGAA
MT	3' biotinylated ssRNA	BLI and fluorescent cleavage assay	CCACAUCUACGAAUUCGUACCAUAGAGAGGUUAUCCGCUC ACCGAGAUCGGAAGAGCACA/3Bio/
C2G	3' biotinylated ssRNA	BLI and fluorescent cleavage assay cleavage assay	CCACAUCUACGAAUUCGUACCAUAGAGAGGUUAUCCGCUG ACCGAGAUCGGAAGAGCACA/3Bio/
C4A	3' biotinylated ssRNA	BLI and fluorescent cleavage assay	CCACAUCUACGAAUUCGUACCAUAGAGAGGUUAUCCGAUC ACCGAGAUCGGAAGAGCACA/3Bio/
G5U	3' biotinylated ssRNA	BLI and fluorescent cleavage assay	CCACAUCUACGAAUUCGUACCAUAGAGAGGUUAUCCUCUC ACCGAGAUCGGAAGAGCACA/3Bio/
C7A	3' biotinylated ssRNA	BLI and fluorescent cleavage assay	CCACAUCUACGAAUUCGUACCAUAGAGAGGUUAUACGCUC ACCGAGAUCGGAAGAGCACA/3Bio/
U10G	3' biotinylated ssRNA	BLI and fluorescent cleavage assay	CCACAUCUACGAAUUCGUACCAUAGAGAGGUGAUCCGCUC ACCGAGAUCGGAAGAGCACA/3Bio/
G15A	3' biotinylated ssRNA	BLI and fluorescent cleavage assay	CCACAUCUACGAAUUCGUACCAUAGAAAGGUUAUCCGCUC ACCGAGAUCGGAAGAGCACA/3Bio/
A18G	3' biotinylated ssRNA	BLI and fluorescent cleavage assay	CCACAUCUACGAAUUCGUACCAUGGAGAGGUUAUCCGCUC ACCGAGAUCGGAAGAGCACA/3Bio/
A20C	3' biotinylated ssRNA	BLI and fluorescent cleavage assay	CCACAUCUACGAAUUCGUACCCUAGAGAGGUUAUCCGCUC ACCGAGAUCGGAAGAGCACA/3Bio/
C22A	3' biotinylated ssRNA	BLI and fluorescent cleavage assay	CCACAUCUACGAAUUCGUAACAUAGAGAGGUUAUCCGCUC ACCGAGAUCGGAAGAGCACA/3Bio/
5'-UAA	3' biotinylated ssRNA	BLI	CCACAUCUACGAAUUCUAACCAUAGAGAGGUUAUCCGCUC ACCGAGAUCGGAAGAGCACA/3Bio/

3'-GCU	3' biotinylated ssRNA	BLI	CCACAUCUACGAAUUCGUACCAUAGAGAGGUUAUCCGCUCAGCUAGAUCGGAAGAGCACA/3Bio/
3'-GGU	3' biotinylated ssRNA	BLI	CCACAUCUACGAAUUCGUACCAUAGAGAGGUUAUCCGCUCAGGUAGAUCGGAAGAGCACA/3Bio/
3'-UAA	3' biotinylated ssRNA	BLI	CCACAUCUACGAAUUCGUACCAUAGAGAGGUUAUCCGCUCAUAAAGAUCGGAAGAGCACA/3Bio/
3'-UGG	3' biotinylated ssRNA	BLI	CCACAUCUACGAAUUCGUACCAUAGAGAGGUUAUCCGCUCAUGGAGAUCGGAAGAGCACA/3Bio/
3'-GUU	3' biotinylated ssRNA	BLI	CCACAUCUACGAAUUCGUACCAUAGAGAGGUUAUCCGCUCAGUUAGAUCGGAAGAGCACA/3Bio/
crRNA-1 target (MT)	ssRNA	Fluorescent cleavage assay	UUGGAAAACUUCAAGAUGUGGUCAACCAAAAUGCACAAGC
crRNA-1 target (D950N)	ssRNA	Fluorescent cleavage assay	UUGGAAAACUUCAAAAUGUGGUCAACCAAAAUGCACAAGC
crRNA-2 target (MT)	ssRNA	Fluorescent cleavage assay	UCCACAGCAAGUGCACUUGGAAAACUUCAAGAUGUGGUCA
crRNA-2 target (D950N)	ssRNA	Fluorescent cleavage assay	UCCACAGCAAGUGCACUUGGAAAACUUCAAAAUGUGGUCA
crRNA-1 target (MT)	3' biotinylated ssRNA	BLI	UUGGAAAACUUCAAGAUGUGGUCAACCAAAAUGCACAAGC/3Bio/
crRNA-1 target (D950N)	3' biotinylated ssRNA	BLI	UUGGAAAACUUCAAAAUGUGGUCAACCAAAAUGCACAAGC/3Bio/
crRNA-2 target (MT)	3' biotinylated ssRNA	BLI	UCCACAGCAAGUGCACUUGGAAAACUUCAAGAUGUGGUCA/3Bio/
crRNA-2 target (D950N)	3' biotinylated ssRNA	BLI	UCCACAGCAAGUGCACUUGGAAAACUUCAAAAUGUGGUCA/3Bio/
Poly-U reporter	5' Fluorescein, 3' quencher ssRNA	Fluorescent reporter for the cleavage assay	6-FAM-UUUUU-Iowa Black FQ
