# Supplementary figures and images for: Massively parallel profiling of RNA-targeting CRISPR-Cas13d
Source: Nat Commun. 2024 Jan 12;15:498. doi: 10.1038/s41467-024-44738-w (PMC10786891; doi:10.1038/s41467-024-44738-w)

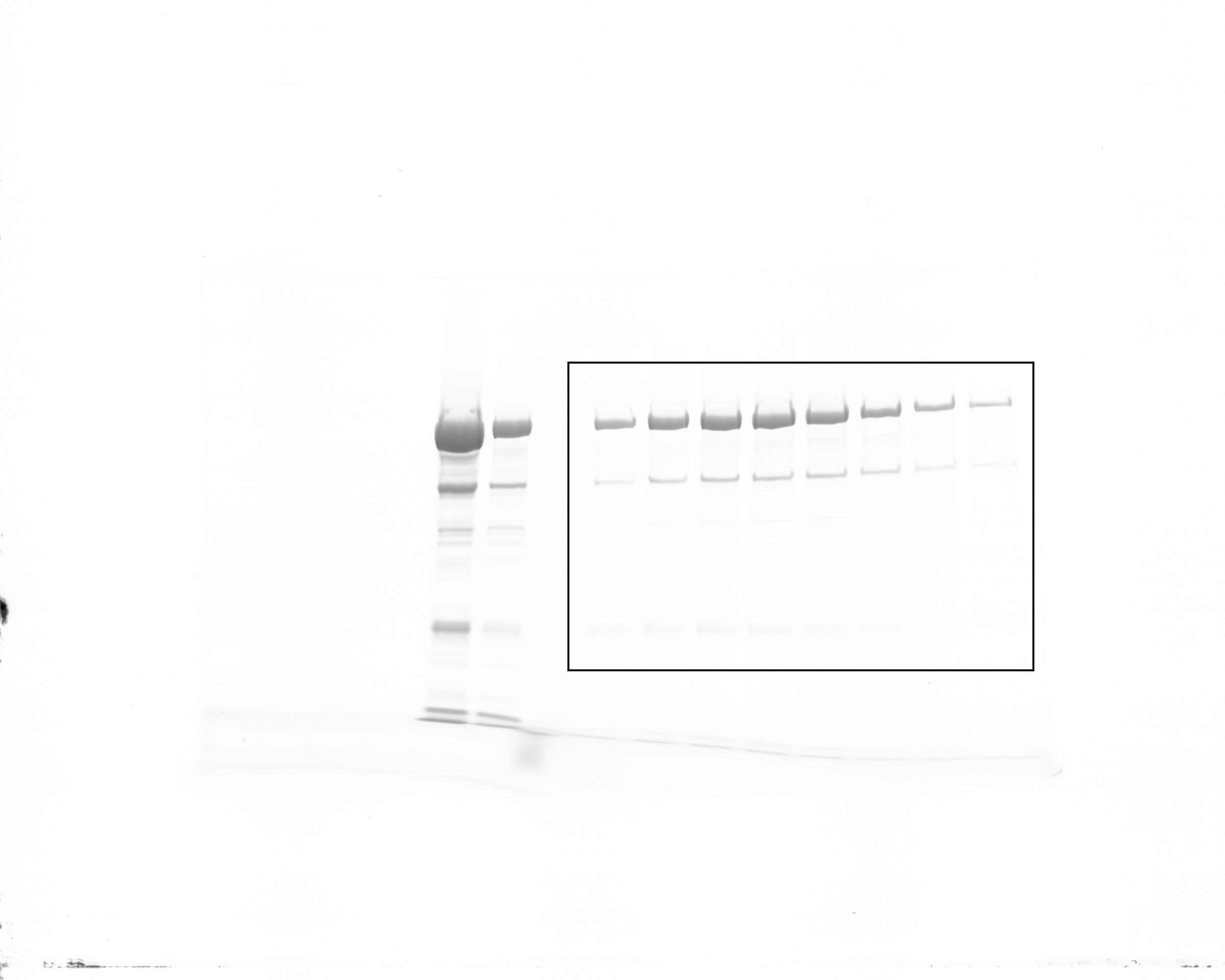

Supplement: Supplementary file 4 — Source Data [file 41467_2024_44738_MOESM4_ESM.zip › Source Data/FigS1C_uncropped_gel_bottom.jpeg]

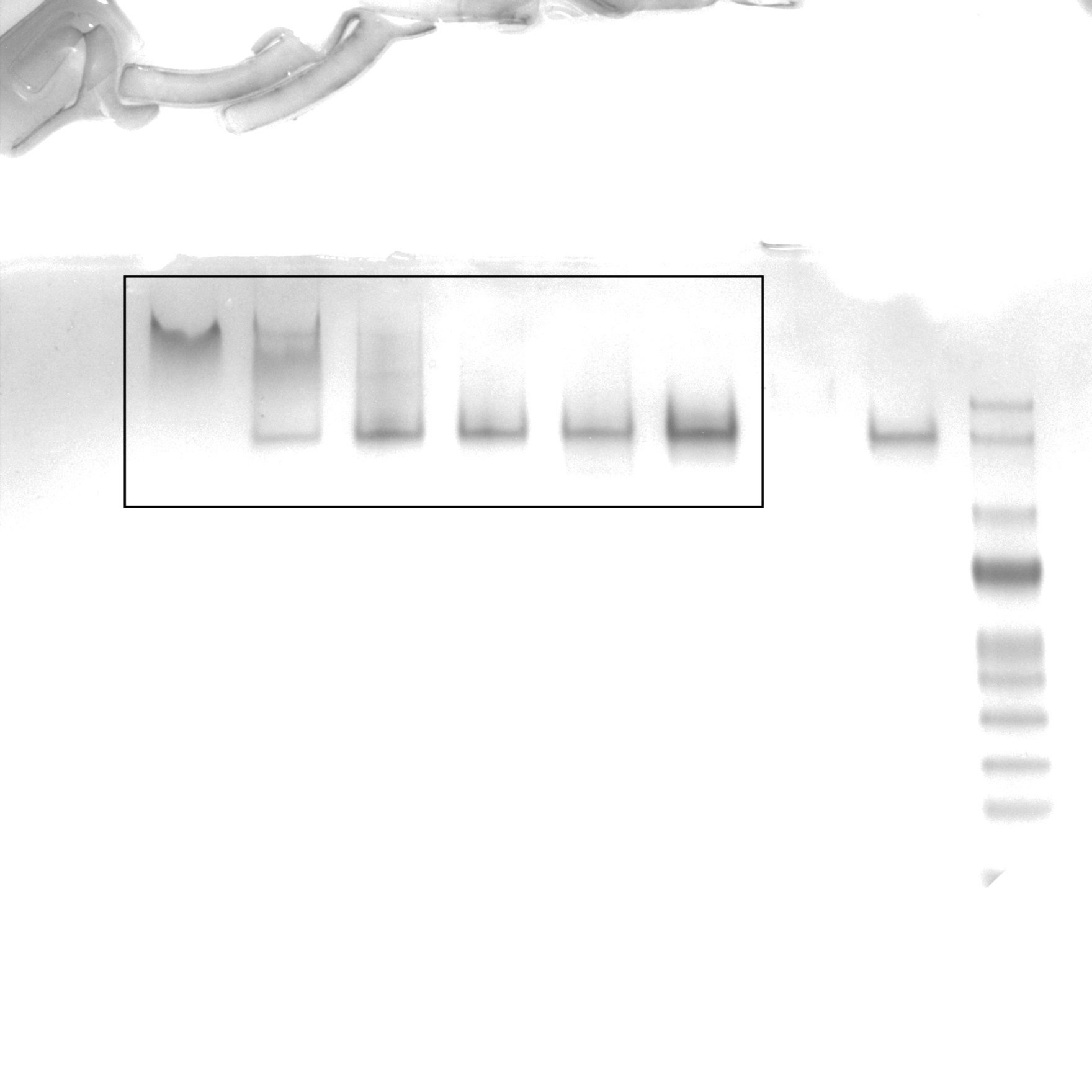

Supplement: Supplementary file 4 — Source Data [file 41467_2024_44738_MOESM4_ESM.zip › Source Data/FigS1D_uncropped_gel.jpg]

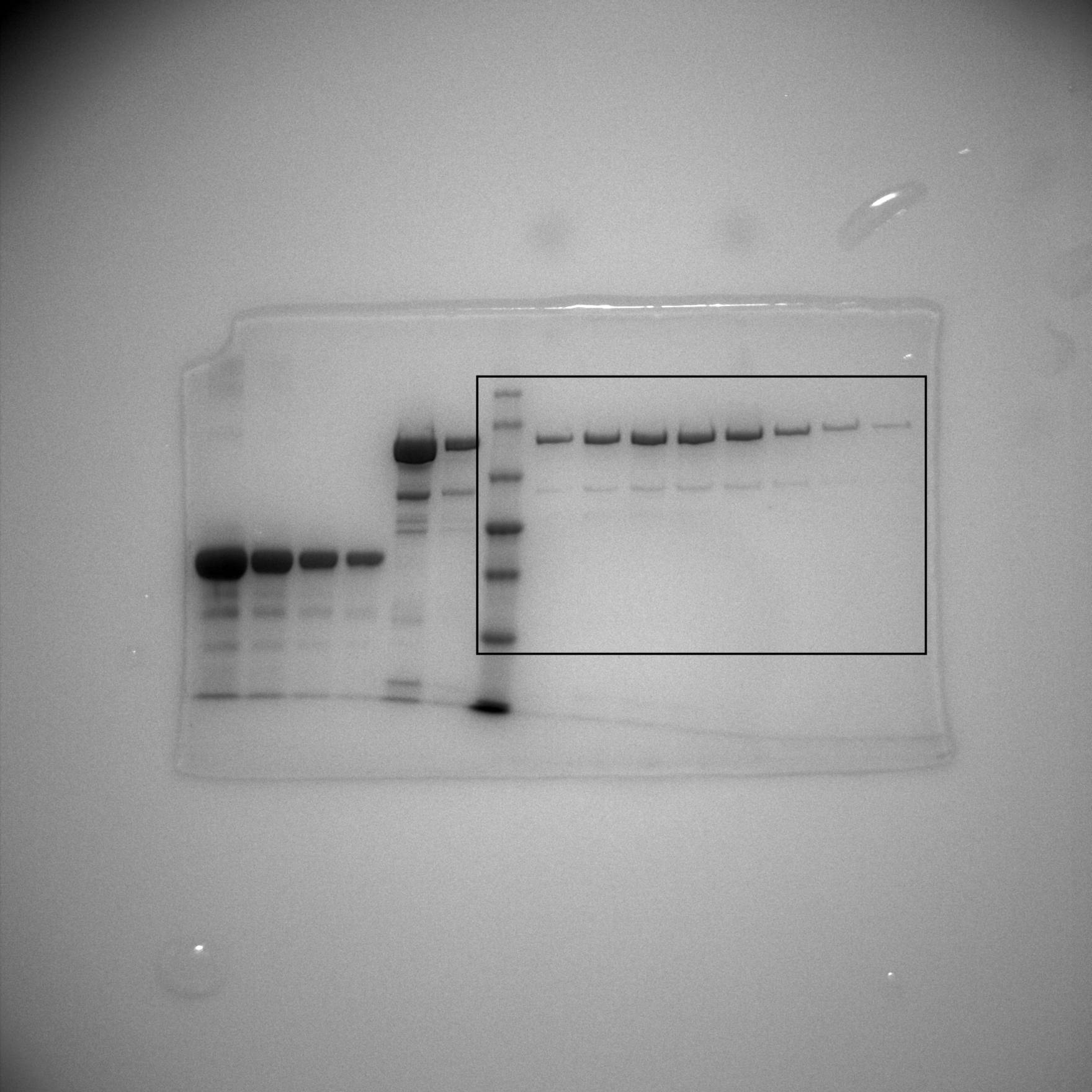

Supplement: Supplementary file 4 — Source Data [file 41467_2024_44738_MOESM4_ESM.zip › Source Data/FigS1C_uncropped_gel_top.tif]
